# Impact of obesity on the gut microbiome and inflammatory markers during SIV infection and antiretroviral therapy

**DOI:** 10.1128/spectrum.00733-25

**Published:** 2025-08-27

**Authors:** Casey M. McGuire, Isaac R. Cinco, Diana Takahashi, Kristin A. Sauter, Melissa Kirigiti, Ilhem Messaoudi, Jonah B. Sacha, Charles T. Roberts, Paul Kievit

**Affiliations:** 1 Division of Metabolic Health and Disease, Oregon National Primate Research Center (ONPRC), Beaverton, Oregon, USA; 2 Department of Microbiology, Immunology, and Molecular Genetics, University of Kentucky 214561, Lexington, Kentucky, USA; 3 Division of Pathobiology and Immunology, ONPRC, Beaverton, Oregon, USA; 4 Division of Reproductive and Developmental Sciences, ONPRC, Beaverton, Oregon, USA; University of Nebraska-Lincoln, Lincoln, Nebraska, USA

**Keywords:** gut microbiome, obesity, human immunodeficiency virus, simian immunodeficiency virus, antiretroviral therapy, inflammation

## Abstract

Dysbiosis and impaired gut barrier integrity contribute to chronic immune activation associated with both obesity and HIV infection. Given the increased incidence of obesity in people living with HIV, we explored the impact of obesity on the gut microbiome and microbial translocation (MT) biomarkers during HIV infection and antiretroviral therapy (ART). Lean and obese rhesus macaques were infected with simian immunodeficiency virus (SIV) and subsequently treated with ART. Obese animals exhibited higher initial MT and inflammation biomarkers that remained constant throughout the study, while lean animals exhibited significant increases in these biomarkers that approached levels observed in obese animals. Lean and obese animals exhibited similar observed amplicon sequence variants (ASVs) at baseline, with obese animals exhibiting reduced ASVs during acute SIV infection that rebounded after 39 weeks of ART treatment. Beta diversity differed between groups and was longitudinally altered in obese animals. Obese animals exhibited significant changes in differential abundance in four times as many bacterial genera compared to lean animals. Our finding that MT and inflammation biomarkers significantly changed in lean animals, while obese animals exhibited significant alterations in microbial diversity, suggests that microbiome changes and systemic inflammation may not directly correlate during SIV infection and ART.

**IMPORTANCE:**

In response to the obesity epidemic, the incidence of obesity at the time of HIV diagnosis has increased, but the impacts of pre-existing obesity throughout antiretroviral therapy (ART)-treated HIV infection remain underexplored. Both obesity and HIV infection have been associated with inflammatory and microbiome perturbations. Here, we utilized 16S rRNA amplicon sequencing and quantification of systemic inflammation markers to longitudinally characterize the fecal microbiome and systemic inflammation in lean and obese rhesus macaques throughout simian immunodeficiency virus (SIV) infection and ART. Lean animals exhibited marked increases in inflammatory markers corresponding with minimal gut microbiome perturbations throughout SIV infection and ART. In contrast, obese animals exhibited minimal inflammatory alterations corresponding with distinct differentially abundant fecal bacterial taxa throughout SIV infection and ART. Our results provide crucial insights into the interactions between pre-existing obesity, inflammation, and the gut microbiome that may aid in developing therapeutic strategies for obese individuals diagnosed with HIV.

## INTRODUCTION

The commensal relationship between a host and its community of gastrointestinal microbes plays a significant role in promoting health and disease. The gut microbiome aids the host in digestion, nutrient extraction, metabolite synthesis, maintenance of the gut mucosal barrier, protection from pathogens, and development/maintenance of innate and adaptive immunity (1). Dysbiosis of the gut microbiome can influence epigenetic modifications and transcription factor binding (2), neurocognitive dysfunction (3), systemic inflammation (4), and metabolite production, and can also exert an effect on conditions such as obesity, insulin resistance, dyslipidemia (5–7), and irritable bowel syndrome (8). The microbiome often exerts its physiological effects via the production of bacterial metabolic products such as short-chain fatty acids (SCFAs) and structural constituents such as lipopolysaccharide (LPS) (1, 9). In altered physiological states, microbial dysbiosis and loss of intestinal barrier integrity can increase the translocation of bacteria and their metabolites from the gut lumen into the circulation, potentially resulting in adverse outcomes. The association of an inflammatory microbiome signature with elevated levels of C-reactive protein (CRP) (10) suggests that excess microbial translocation (MT) can activate the host immune system, resulting in increased systemic inflammation. Increased translocation of bacterial LPS activates the immune system through its binding to LPS binding protein (LBP) and interaction with CD14, which has been associated with elevated production of other inflammatory biomarkers such as CRP (11). Therefore, systemic measurements of LBP, soluble CD14 (sCD14), and CRP can be used to indicate the degree of LPS translocation and, therefore, immune activation (12). As an interface between health and disease, it is important to understand how the microbiome affects and is altered by various conditions to develop potential personalized treatments for microbiome-associated physiological conditions.

Many studies have described the relationship between the gut microbiome and obesity. Obesity, metabolic syndrome, and impaired glucose homeostasis have been associated with an overall decrease in microbial diversity, particularly the abundance of bacterial species that produce SCFAs and promote gut barrier integrity (13). An obesogenic gut microbiome phenotype has been reported to consist of an increased relative abundance of Firmicutes, Fusobacteria, and Proteobacteria and decreased relative abundance of the Verrucomicrobiota, Faecalibacterium, and Bacteroidetes phyla (14), as well as a decreased relative abundance of *Catenibacterium*, *Dialister*, *Dorea*, *Megasphaera*, *Prevotella*, *Streptococcus*, and *Sutterella* genera (15). The obese microbiome is associated with an increased capacity for energy harvest, specifically increased carbohydrate metabolism and absorption that may play a key role in diet-induced adiposity (16). In the context of obesity-associated microbiome dysbiosis, gut barrier integrity often degrades, leading to increased MT of bacteria and their metabolites, including SCFAs and LPS, followed by subsequent immune activation associated with unhealthy metabolic profiles (12, 17). As a result, obese individuals exhibit higher levels of systemic markers that indicate a leaky gut, including zonulin, LBP, and sCD14 (18), as well as increased levels of fecal and circulating SCFAs (19). Inflammation may also occur in individuals with obesity due to LPS-induced adipocyte pyroptosis, as larger adipocytes are more prone to it (20).

As HIV infection targets the gut mucosa and depletes the resident CD4+ T cell, antigen-presenting cell, and innate lymphocyte populations, increased immune activation in HIV-infected individuals has also been linked to gut microbiome dysbiosis and MT (21). There have been mixed reports regarding the impact of HIV infection on the diversity of the gut microbiome, with some studies reporting no change and others reporting decreased diversity (22). HIV-infected individuals have been shown to exhibit changes in the intestinal microbiome, which may be associated with disease progression (22–24). Alterations in gut microbiome composition as a result of HIV infection, such as increased *Prevotella*, *Fusobacteria,* and *Anaerococcus* and depleted *Roseburia*, *Coprococcus*, *Ruminococcaceae*, *Clostridia*, *Akkermansia*, and *Lachnospira* genera, often persist even with viral loads suppressed with antiretroviral therapy (ART) and potentially play a role in the development of systemic inflammation, indicated by markedly higher circulating sCD14 (25–28). Previous studies utilizing the simian immunodeficiency virus (SIV)-infected macaque model of HIV have also reported alterations in the gut microbiome and virome (29–31). It has also been shown that ART administration in both infected and uninfected humans and macaques impacts microbial diversity and composition but fails to fully restore the state of the microbiome after infection (32–36).

Certain ART regimens themselves are associated with increased metabolic complications, including lipodystrophy, hyperlipidemia, cardiovascular disease, and impaired glucose tolerance in people living with HIV. The higher risk of metabolic comorbidities in people with HIV, even with viral levels controlled by ART, may be associated with gut microbiota changes that occur following HIV infection and ART (37–39). This may be further exacerbated by pre-existing obesity and metabolic comorbidities at the time of diagnosis, as well as an obesogenic western-style diet (WSD) and its effects on the microbiome (40–44).

Previous studies utilizing a SIV-infected macaque model of HIV indicated that short-term exposure to a WSD exacerbated the pathogenesis and progression of SIV/HIV infection in the absence of ART (45, 46), but did not assess the potential impact that established pre-existing obesity and metabolic dysfunction, which increasingly affects people at the time of HIV diagnosis (47–49), may have on HIV pathogenesis and microbiome-mediated interactions, or the effect of subsequent long-term ART. In the present study, we employed a preclinical SIV-infected rhesus macaque model of HIV. This model is not confounded by lifestyle risk factors associated with HIV acquisition in the human population, to assess the correlations between metabolic status (established long-term obesity), SIV/HIV infection, ART, microbiome composition, and physiological parameters of inflammation (50, 51). A better understanding of the interplay between microbiome-mediated energy homeostasis and immune activation in SIV/HIV infection and ART between lean and obese individuals is crucial in designing strategies for preventing and managing systemic inflammation and metabolic comorbidities in obese individuals diagnosed with HIV (52, 53).

## RESULTS

**Fig 1:**
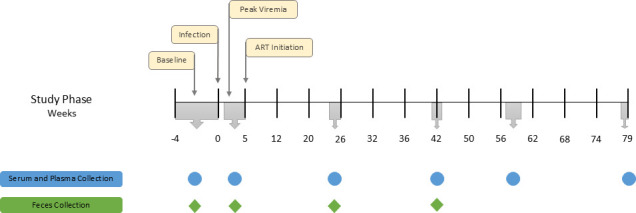
Experimental timeline. The gray boxes on the timeline indicate the period over which samples were taken for each time point.

To explore the effects of diet-induced obesity on the gut microbiome’s response to SIV infection and long-term ART, we infected lean and obese rhesus macaques with SIVmac239M, allowed infection to proceed for 5 weeks, and then initiated daily ART for 74 weeks. Figure 1 depicts the longitudinal experimental design and sampling schedule. Since animals in the obese group had been exposed to a WSD for an extended period prior to infection and ART treatment, we are not able to unequivocally ascribe the baseline differences in levels of inflammatory biomarkers or microbiome profiles described below to diet *per se* versus diet-induced obesity.

### Markers of inflammation and microbial translocation

**Fig 2:**
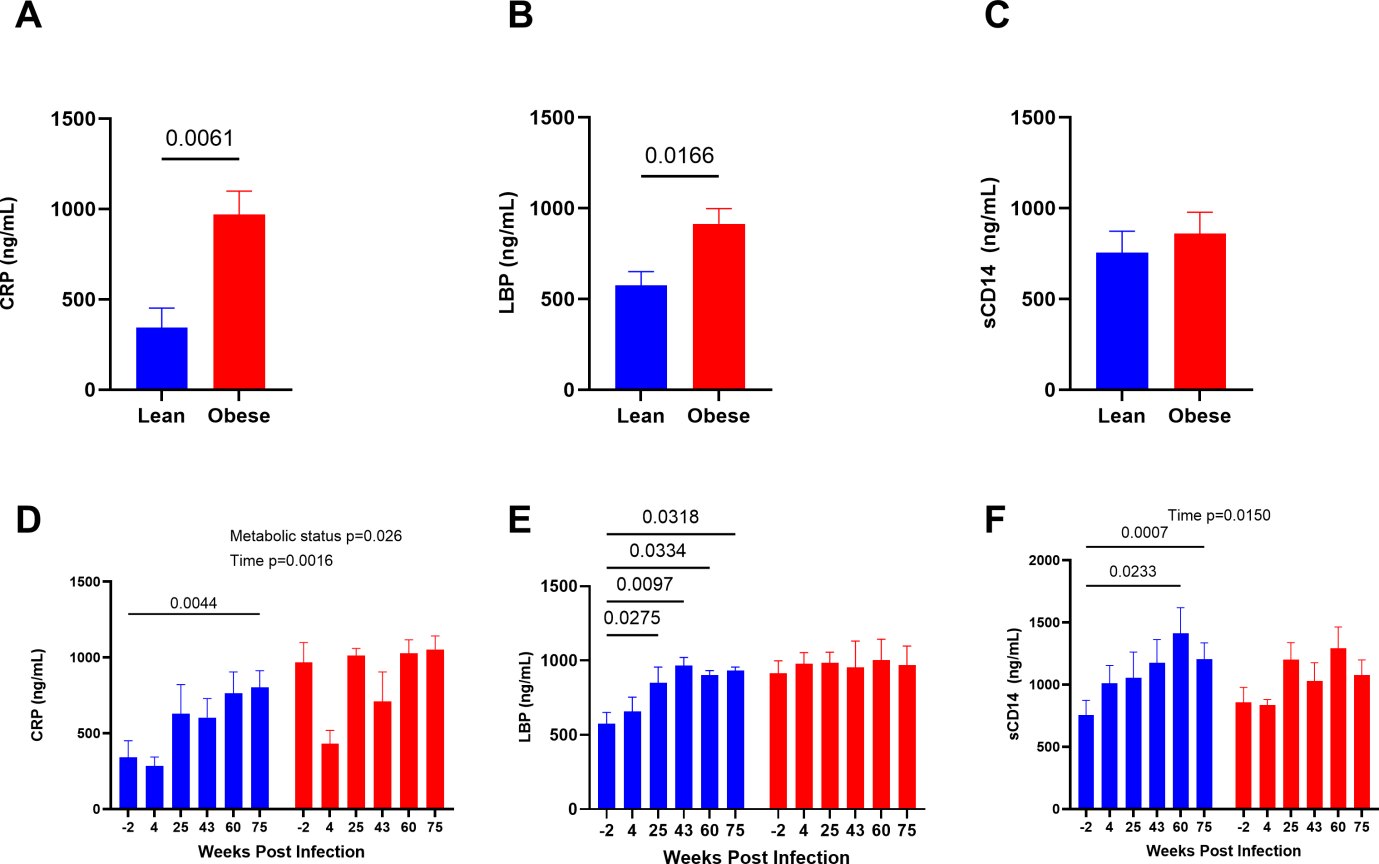
Markers of inflammation and microbial translocation longitudinally increase in lean, but not obese, animals. Baseline (top) and longitudinal (bottom) plasma CRP (**A, D**), LBP (**B, E**), and serum sCD14 (**C, F**) determined by enzyme-linked immunosorbent assay (ELISA). The data shown represents a subset of animals previously described (54). Baseline significance was determined using unpaired *t*-tests. Significance of longitudinal changes in CRP, LBP, and sCD14 was determined using two-way analysis of variance with Dunnett’s multiple comparisons test.

At baseline, plasma CRP (Fig. 2A) and serum LBP (Fig. 2B) levels were significantly higher in obese animals compared to lean animals. Following SIV infection and ART treatment, lean animals exhibited significant increases in CRP (Fig. 2D) and LBP (Fig. 2E) that ultimately reached levels equivalent to those of obese animals. sCD14 levels were not significantly different between lean and obese animals at baseline (Fig. 2C). Lean animals exhibited significant increases in sCD14 throughout SIV infection and ART when compared to baseline (Fig. 2F). For all measurements, obese animals did not show any significant changes throughout the time course (Fig. 2D through F).

### Taxonomic composition

**Fig 3:**
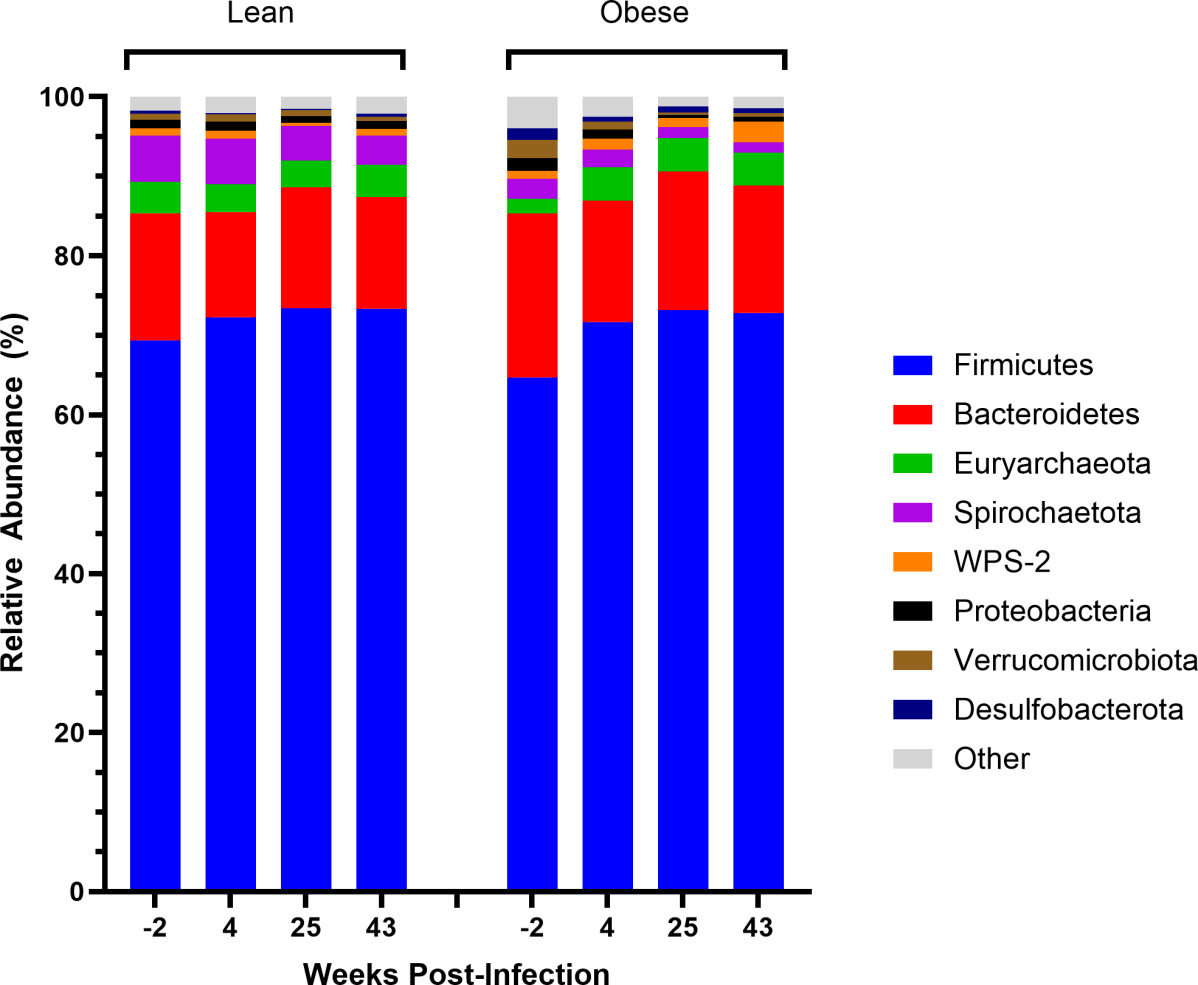
Profiles of bacterial phyla throughout SIV Infection and ART. Stacked bar plot of abundant phyla organized by group and time point. All phyla averaging less than 1% relative abundance in both lean and obese animals at baseline were grouped into “other.” Bars represent averages for the indicated time point.

**Fig 4:**
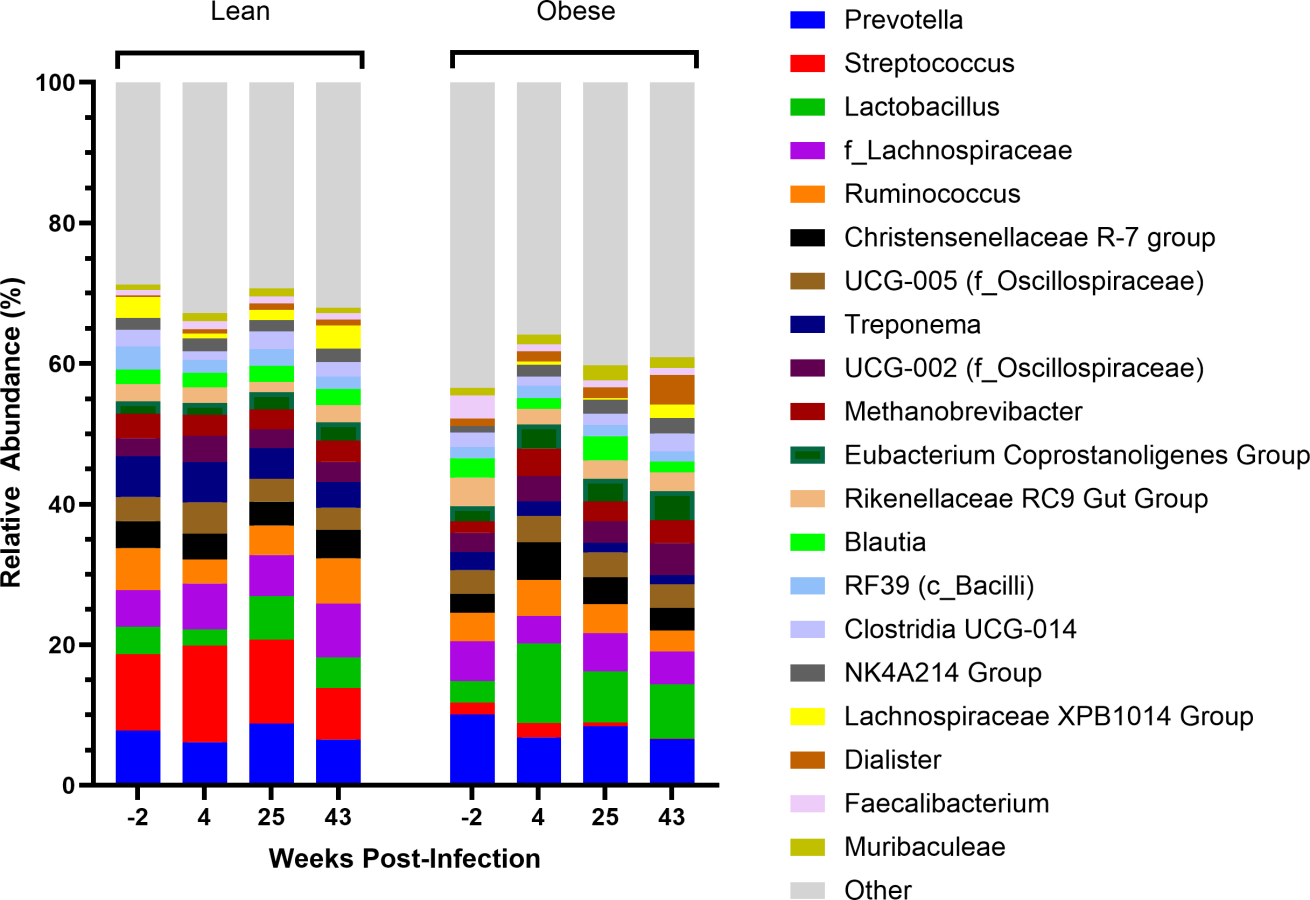
Profiles of bacterial genera throughout SIV Infection and ART. Stacked bar plots of abundant genera organized by group and time point. All genera averaging 1.1% or lower relative abundance were grouped into the “other” category. Bars represent averages for the indicated time point.

In order of most to least abundant, both lean and obese fecal microbiomes were defined by the Firmicutes, Bacteroidetes, Euryarchaeota, and Spirochaetota phyla, which in total accounted for 94.7%–96.3% abundance in lean animals and 89.7%–96.2% abundance in obese animals throughout the time course. Both groups exhibited lesser prevalence of WPS-2, Proteobacteria, Verrucomicrobiota, Desulfobacterota, and other phyla which averaged less than 1% relative abundance in both lean and obese animals at baseline (Fig. 3). At all time points, lean animals demonstrated a trend toward increased relative abundance of the Spirochaetota phylum compared to obese animals. Both lean and obese animals exhibited a modest shift toward increased Firmicutes and decreased Bacteroidetes during SIV infection, and that trend remained even after full viral suppression (Fig. 3). Obese animals had a longitudinal increase in WPS-2 and Euryarchaeota accompanied by a decrease of Verrucomicrobiota compared to lean animals (Fig. 3). At the genus level (Fig. 4), lean animals exhibited a trend toward greater relative abundance of *Streptococcus*, *Treponema*, and *Lachnospiraceae XBP1014 group* at all time points when compared to obese animals. Obese animals exhibited an increase in other genera at all time points when compared to lean animals, as well as a longitudinal increase in *Lactobacillus* throughout SIV infection and ART. The other genera averaged 1.1% or lower relative abundance, differentiated by each time point.

### Microbial diversity

**Fig 5:**
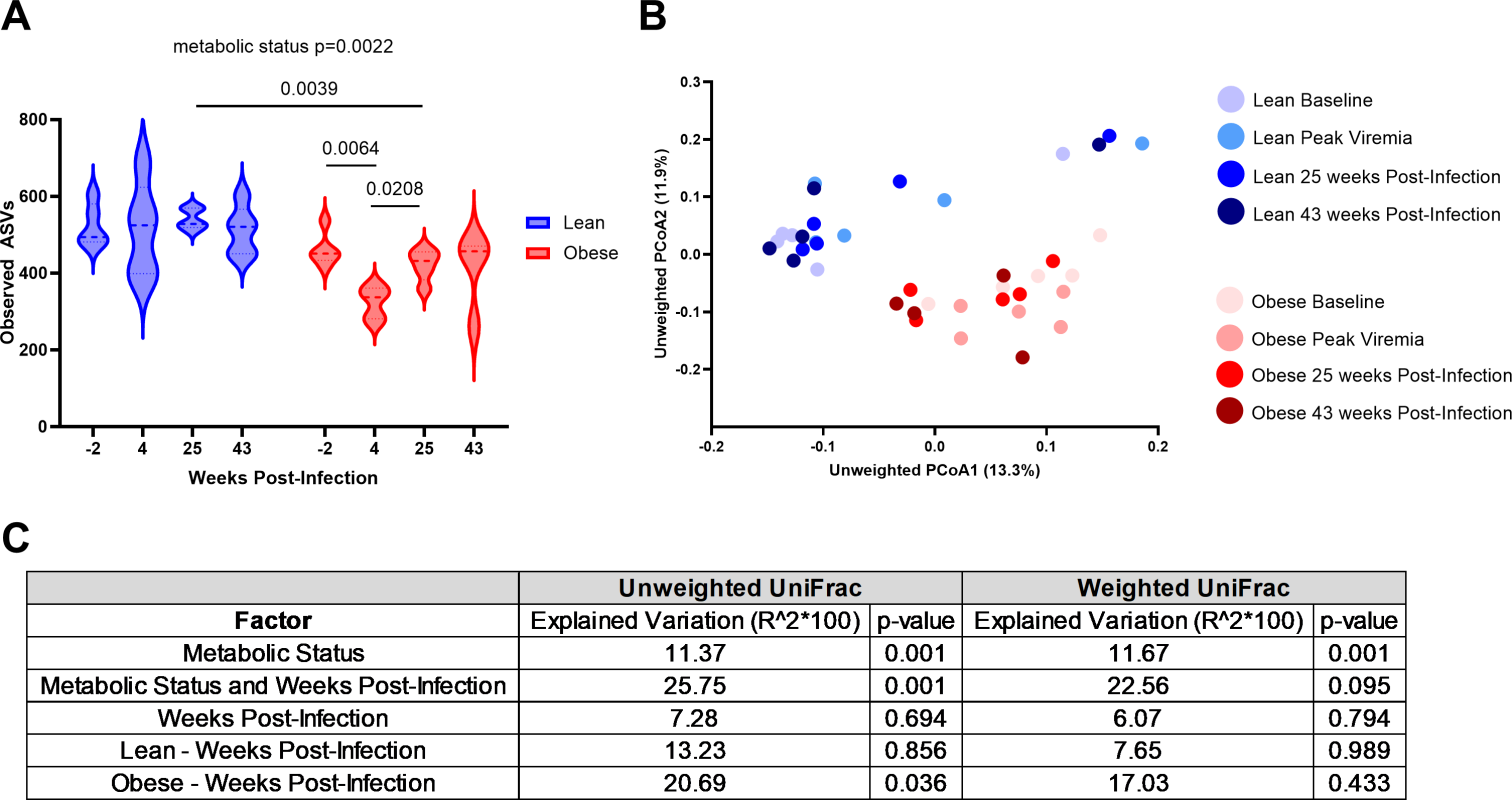
Evolution of lean and obese microbiomes throughout SIV infection and ART. Violin plot of observed ASVs (**A**). After passing a normality/lognormality test, ASV significance was determined using a mixed-effects analysis with Šídák’s multiple comparisons test (between groups) and Tukey’s multiple comparisons test (within groups) (**A**). *, *P* < 0.05; **, *P* < 0.01; ##, *P* < 0.01. Principal coordinate analysis (PCoA) of unweighted UniFrac distances between microbial communities in lean and obese animals throughout SIV infection and ART (**B**). The contribution of metabolic status, weeks post-infection, and both metabolic status and weeks post-infection to the total variance in the unweighted and weighted UniFrac dissimilarity matrices measured using permutational multivariate analysis of variance (PERMANOVA) (Adonis with 10,000 permutations) (**C**). The contribution of weeks post-infection to the total variance in the unweighted and weighted UniFrac dissimilarity matrices within lean and obese groups measured using PERMANOVA (Adonis with 10,000 permutations) (**C**).

Obese animals exhibited a significant depletion in the number of observed amplicon sequence variants (ASVs), indicating a reduced alpha diversity during SIV infection that remained suppressed compared to lean animals until at least 20 weeks after ART initiation (Fig. 5A), at which point animals had reached full viral suppression (54). After 38 weeks of ART, ASVs in obese animals had returned to baseline levels. Lean animals did not exhibit any significant alterations to the number of observed ASVs during the time course, but they did exhibit the largest spread in distribution during acute SIV infection at 4 weeks post-infection (Fig. 5A). Observed ASVs significantly differed between lean and obese animals only at 25 weeks post-infection, with lean animals exhibiting greater numbers of observed ASVs (Fig. 5A). The same trend was observed using the Shannon Evenness and Faith’s phylogenetic diversity alpha diversity metrics, wherein obese animals exhibited decreased evenness and diversity during acute SIV infection (Fig. S1A) as well as decreased phylogenetic diversity in obese animals compared to lean animals at baseline and 25 weeks post-infection (Fig. S1B).

A principal coordinate analysis (PCoA) of unweighted UniFrac distances between microbial communities indicated that the overall composition of the fecal microbiome in lean and obese animals was distinct from one another throughout SIV infection and ART (Fig. 5B). The PCoA of weighted UniFrac distances between microbial communities demonstrates how samples cluster when relative abundance is considered, indicating that lower-abundance taxa may be a significant source of varying diversity between groups (Fig. S1C). Because we collected samples from lean and obese groups throughout SIV infection and ART, we determined the contribution of metabolic status and weeks post-infection (i.e., duration of ART) to the variation within UniFrac dissimilarity matrices using a permutational multivariate analysis of variance (PERMANOVA) (Fig. 5C). This analysis revealed that metabolic status explained a significant amount of the total variation (11.37%–11.67%, *P* = 0.001) compared to weeks post-infection (6.07%–7.28%, *P* > 0.05). Combined metabolic status and weeks post-infection explained a significant amount of the total variation in the unweighted UniFrac analysis (25.5%, *P* = 0.001), but not in the weighted UniFrac analysis (22.56%, *P* = 0.095). Also using PERMANOVA, we assessed the contribution of weeks post-infection on the total variation within UniFrac dissimilarity matrices within lean and obese groups themselves. We determined that the number of weeks post-infection explained a significant amount of variation within the unweighted UniFrac dissimilarity matrix in obese animals (20.69%, *P* = 0.036), but not within the weighted UniFrac dissimilarity matrix (17.03%, *P* = 0.433). Weeks post-infection did not explain a significant amount of total variation in lean animals (7.65%–13.23%, *P* > 0.05) (Fig. 5C).

### Differentially abundant genera between lean and obese animals

**Fig 6:**
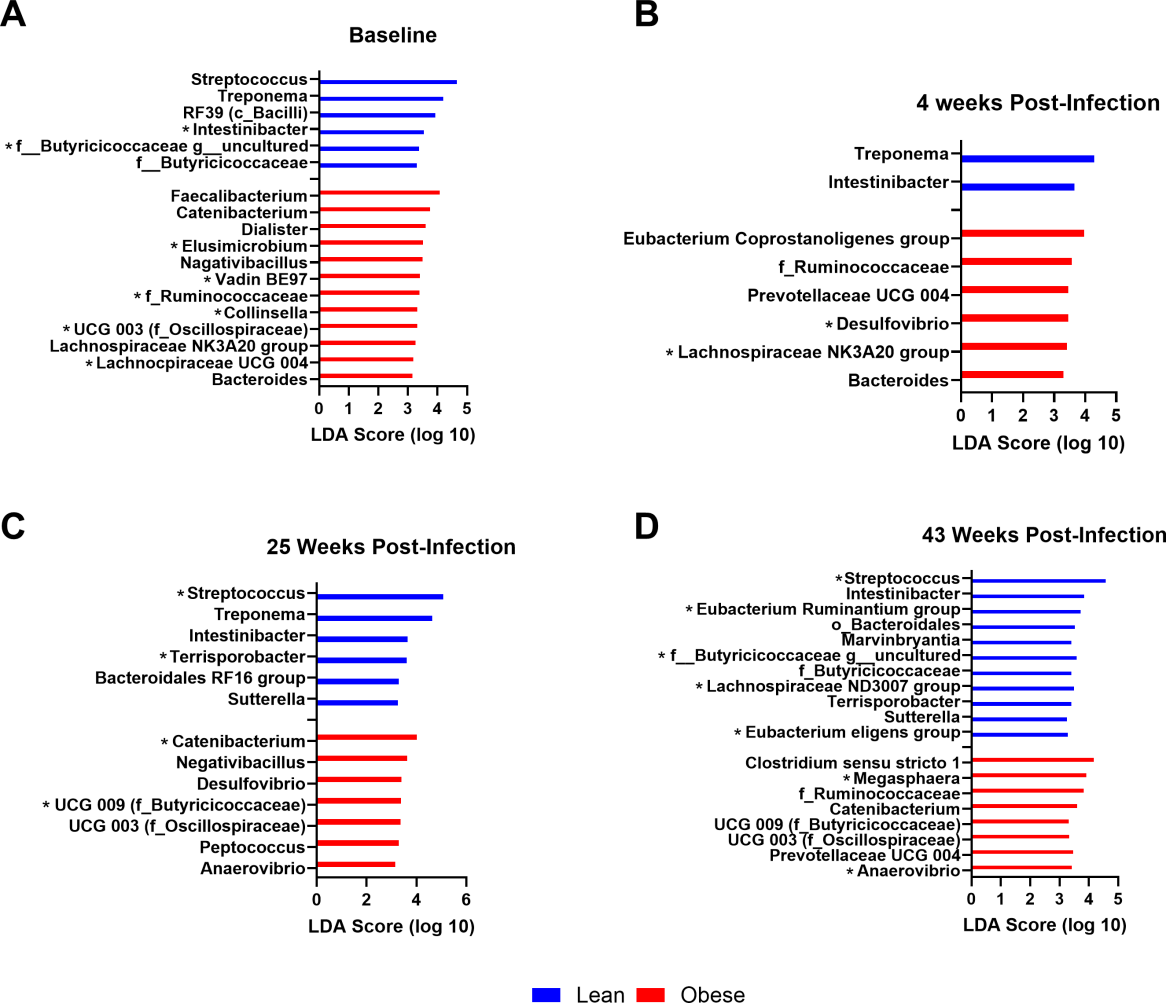
Differentially abundant genera between lean and obese animals throughout SIV infection and ART determined by LEfSe analysis. LEfSe demonstrating differentially abundant genera between lean and obese animals at baseline (**A**), 4 weeks post-infection (**B**), 25 weeks post-infection (**C**), and 43 weeks post-infection (**D**). When generating the LEfSe (log10 linear discriminant analysis [LDA] score >2), the time point was used as the subject while the condition (lean or obese) was used as the class. Taxa marked with an asterisk (*) were found to be differentially abundant by both LEfSe and analysis of compositions of microbiomes with bias correction (ANCOM-BC) analyses at one or more time points.

The linear discriminant analysis effect size (LEfSe) algorithm (55) was used to determine which genera were driving the observed differences in microbiome composition between lean and obese animals at each time point throughout the study (Fig. 6).

Lean animals exhibited greater relative abundance of *Intestinibacter* than obese animals throughout the time course (Fig. 6A through D). Lean animals also had a greater relative abundance of *Treponema* at all time points except for 43 weeks post-infection (Fig. 6A through C). *Sutterella* and *Terrisporobacter* were more abundant in lean animals during ART treatment at 25 and 43 weeks post-infection (Fig. 6C and D).

Obese animals exhibited greater relative abundance of *Catenibacterium* than lean animals at all time points, except during acute SIV infection at 4 weeks post-infection (Fig. 6A, C, and D). The family Ruminococcaceae was more abundant in obese animals at all time points except for 25 weeks post-infection (Fig. 6A, B, and D). Obese animals also exhibited greater relative abundance of *Bacteroides* and *Lachnospiraceae NK3A20 group* at baseline and 4 weeks post-infection (Fig. 6A and B), *Negativibacillus* at baseline and 25 weeks post-infection (Fig. 6A and C), and *Desulfovibrio* at 4 and 25 weeks post-infection (Fig. 6B and C).

We also performed analysis of compositions of microbiomes with bias correction (ANCOM-BC) as a secondary method to assess differentially abundant ASVs between lean and obese animals at each time point. Many of the genera identified by LEfSe were also identified by ANCOM-BC at one or more time points, such as *Desulfovibrio* during acute SIV infection and both *Megasphaera* and *Anaerovibrio* at 43 weeks post-infection in obese animals, as well as *Streptococcus* in lean animals at 25 and 43 weeks post-infection (Fig. 6B and D; Fig. S2B and D). ANCOM-BC analysis identified a few additional genera as differentially abundant as well, such as *Victivallis* in obese animals at baseline (Fig. S2A), *Tuzzerella* in lean animals during acute SIV infection (Fig. S2B), and *Defluviitaleaceae UCG-001* in lean animals during ART at 25 and 43 weeks post-infection (Fig. S2C and D). Bacterial genera identified as differentially abundant by both LEfSe and ANCOM-BC analyses are marked with an asterisk (*) in Fig. 6; Fig. S2.

### Differentially abundant genera throughout SIV infection and ART

**Fig 7:**
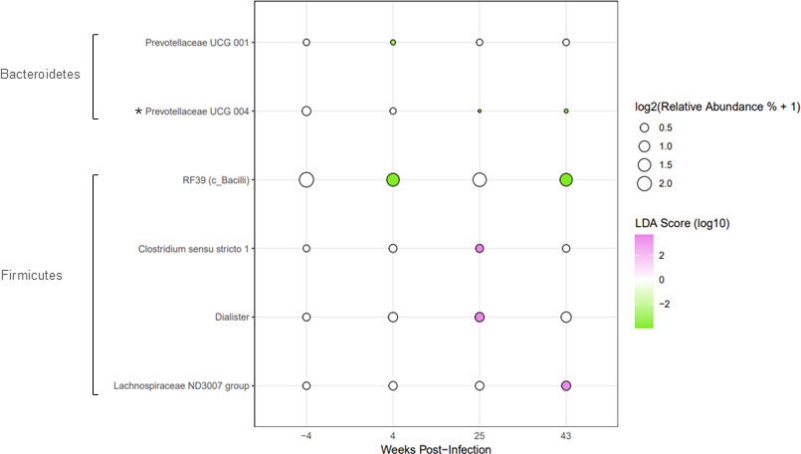
Differentially abundant genera in lean animals throughout SIV infection and ART determined by LEfSe analysis. Bubble plot of LEfSe results illustrating differentially abundant genera in lean animals at 4, 25, and 43 weeks post-infection when compared to baseline (week −4). When generating the LEfSe (log10 LDA score >2), condition (lean) was used as the subject, and time point was used as the class. Taxa marked with an asterisk (*) were found to be differentially abundant by both LEfSe and ANCOM-BC analyses at one or more time points.

**Fig 8:**
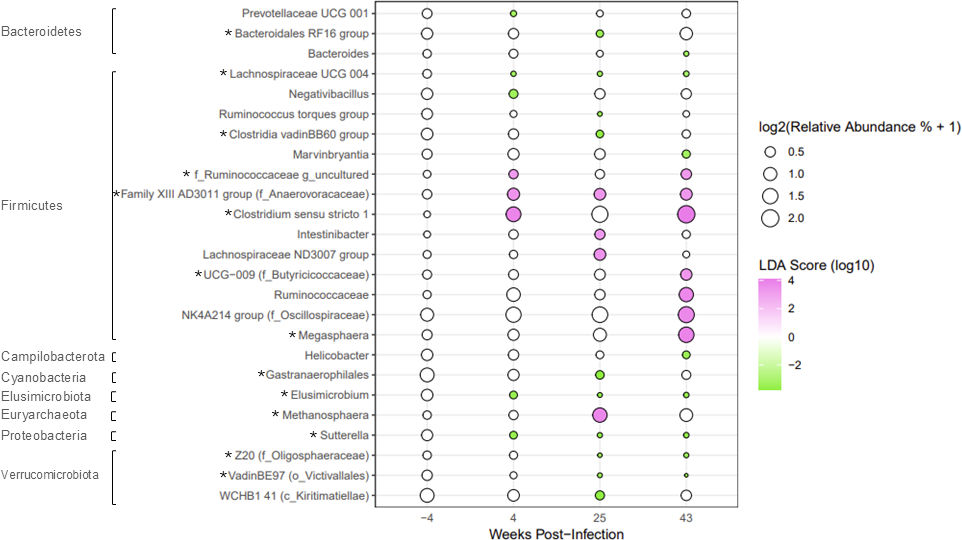
Differentially abundant genera in obese animals throughout SIV infection and ART determined by LEfSe analysis. Bubble plot of LEfSe results determining differentially abundant genera in obese animals at 4, 25, and 43 weeks post-infection when compared to baseline (week −4). Bacterial phyla are indicated by the labeled brackets. When generating the LEfSe (log10 LDA score >2), the condition (obese) was used as the subject, and the time point was used as the class. Taxa marked with an asterisk (*) were found to be differentially abundant by both LEfSe and ANCOM-BC analyses at one or more time points.

After assessing the differential abundance of genera between lean and obese animals throughout SIV infection and ART, we performed a LEfSe analysis (55) to assess the differential abundance of genera within each group throughout the time course (Fig. 7 and 8). Compared to obese animals, lean animals exhibited fewer genera that were significantly altered from baseline throughout SIV infection and ART (Fig. 7 vs Fig. 8). The decrease in *Prevotellaceae UCG 001* seen during acute SIV infection at 4 weeks post-infection was reversed by ART (Fig. 7). Bacilli *RF39* decreased at 4 weeks post-infection and returned to baseline levels at 25 weeks post-infection, coincident with almost complete suppression of plasma viremia, then decreased below baseline levels once again at 43 weeks post-infection. In addition, *Clostridium sensu stricto 1* and *Dialister* were increased at 25 weeks post-infection but returned to baseline levels by 43 weeks post-infection. *Lachnospiraceae ND3007 group* abundance was decreased at 43 weeks post-infection, while *Prevotellaceae UCG 004* abundance was decreased at both 25 and 43 weeks post-infection (Fig. 7).

Obese animals exhibited four times as many differentially abundant bacterial genera and phyla when compared to lean animals throughout SIV infection and ART (Fig. 8) and displayed a more pronounced dysbiosis. *Lachnospiraceae UCG 004*, *Elusimicrobium*, and *Sutterella* decreased with SIV infection and remained decreased throughout ART. *Prevotellaceae UCG 001* and *Negativibacillus* decreased with SIV infection and were attenuated by ART. Early-stage ART corresponded with a decrease in *Bacteroidales RF16 group*, *Ruminococcus torques group*, *Clostridia vadinBB60 group*, *Gastranaerophilales*, and Kiritimatiellae *WCHB 41* at 25 weeks post-infection that returned to baseline levels by 43 weeks post-infection. Oligosphaeraceae *Z20* and Victivallales *VadinBE97* abundance decreased with ART at both 25 and 43 weeks post-infection. *Bacteroides*, *Marvinbryantia*, and *Helicobacter* abundance were decreased at 43 weeks post-infection (Fig. 8).

All but one of the bacteria that increased from baseline in obese animals belonged to the Firmicutes phylum (Fig. 8). Anaerovoracaceae *Family XIII AD3011* group increased during acute SIV infection at 4 weeks post-infection and remained increased throughout treatment with ART. Ruminococcaceae *uncultured* and *Clostridium sensu stricto 1* were more abundant at 4 and 43 weeks post-infection. Higher abundance of *Intestinibacter*, *Lachnospiraceae ND3007 group*, and *Methanosphaera* was observed at 25 weeks post-infection, but returned to baseline levels at 43 weeks post-infection. Increased abundance of Butyricicoccaceae *UCG-009*, *Ruminococcaceae*, *Oscillospiraceae NK4A214 group*, and *Megasphaera* was seen at 43 weeks post-infection (Fig. 8).

Lean and obese animals shared some significant alterations to the differential abundance of certain genera throughout SIV infection and ART (Fig. 7 and 8). Both lean and obese animals exhibited decreased *Prevotellaceae UCG 001* during acute SIV infection at 4 weeks post-infection, which was attenuated by ART. *Clostridium sensu stricto 1* and *Lachnospiraceae ND3007 group* increased in both lean and obese animals at one or more time points throughout the time course. All bacterial genera that increased from baseline in either lean or obese animals belong to the Firmicutes phylum, except for an increase in *Methanosphaera* belonging to the Euryarchaeota phylum at 25 weeks post-infection in obese animals. All Bacteroidetes genera that changed throughout the time course were decreased from baseline in both lean and obese animals. At 43 weeks post-infection, lean animals had only three bacteria that were significantly differentially abundant from baseline compared to obese animals that exhibited 15, indicating a lasting impact on the fecal microbiome following SIV infection and ART in obese animals (Fig. 7 and 8).

We also performed ANCOM-BC analyses to assess differentially abundant genera within lean and obese groups throughout SIV infection and ART. Many of the same genera were identified by both LEfSe and ANCOM-BC algorithms (Fig. S3 and S4). Both algorithms indicated that obese animals exhibited four times as many differentially abundant genera throughout SIV infection and ART compared to lean animals, and that obese animals displayed a larger lasting impact of SIV infection and ART, indicated by the larger number of genera that were differentially abundant at 43 weeks post-infection. Both analyses identified a decrease in Prevotellaceae UCG 004 in lean animals during ART at 25 and 43 weeks post-infection (Fig. 7; Fig. S3), and similar changes in many genera, such as *Bacteroidales RF16 Group*, *Elusimicrobium*, *Methanosphaera*, *Clostridium sensu stricto 1*, *Megasphaera*, and *Sutterella*, throughout the time course in obese animals (Fig. 8; Fig. S4). ANCOM-BC analyses showed that both lean and obese animals exhibited an increase in *Olsenella* during early ART at 25 weeks post-infection (Fig. S3 and S4). Less abundant taxa, such as *Staphylococcus* or *Tuzzerella* in obese animals, were shown to be differentially abundant throughout the time course by ANCOM-BC only (Fig. S4), likely having been filtered out due to low abundance prior to assessment using the LEfSe algorithm. Bacterial genera identified as differentially abundant by both LEfSe and ANCOM-BC analyses are marked with an asterisk (*) in Fig. 7 and 8; Fig. S3 and S4.

## DISCUSSION

This study sought to assess the impact of pre-existing obesity and metabolic dysfunction on the microbiome, MT, and systemic inflammation throughout SIV infection and long-term ART. By utilizing obese NHPs with an established history of WSD consumption, low-grade inflammation, and insulin resistance, we were able to differentially compare the effects of pre-existing obesity, SIV infection, and ART on both the microbiome and inflammatory markers associated with MT. Our data reveal distinct trends in microbial diversity and inflammatory biomarker responses throughout infection and ART in lean and obese subjects.

Despite similar microbial richness at baseline as indicated by ASV counts, only obese animals exhibited a significant depletion in the number of observed ASVs during acute SIV infection, which rebounded at 25 weeks post-infection after ART initiation but was significantly lower than that of the number of ASVs observed in lean animals. Significant alterations in the number of observed ASVs, as well as depletion of beta diversity, throughout the time course in obese but not lean animals suggest that obesity decreases the ability to maintain microbial diversity throughout SIV infection and ART. Interestingly, only lean animals exhibited significant increases in markers of MT and inflammation throughout the experimental time course, which contrasts with a previous report that found that plasma lipopolysaccharide was reduced back to normal levels upon ART initiation, and no changes in circulating levels of sCD14 were observed (56).

The microbiome of obese animals was more susceptible to the impact of SIV infection and ART treatment, indicated by the greater number of genera that were significantly altered throughout the time course. Consistent with their dietary intake and metabolic phenotype, obese animals exhibited a significantly greater relative abundance of the *Bacteroides* genus compared to lean animals at baseline and during acute SIV infection. The *Bacteroides* genus is associated with a diet high in animal fat and is increased in people with metabolic syndrome and non-alcoholic fatty liver disease (NAFLD) (57). The significant decrease in *Bacteroides* observed in obese animals at 43 weeks post-infection could be a result of decreased appetite, an altered need for energy harvest, or an improvement in insulin resistance due to weight loss throughout the time course as previously described (54). An increase in the Gram-negative sulfate-reducing bacterial genus *Desulfovibrio* is also observed in metabolic syndrome, WSD-fed animal models, type 2 diabetes, NAFLD (58, 59), and ulcerative colitis mucosal inflammation (60). We found a greater abundance of this genus in obese animals during acute SIV infection and early ART versus lean animals. The genus *Tuzzerella*, associated with diets high in fat and sugar and positively correlated with metabolic disorders (61, 62), was transiently more abundant in lean animals compared to obese animals during acute SIV infection and was also increased during long-term ART compared to baseline in obese animals. Alterations in genera associated with dietary habits and metabolic disorders in lean and obese animals throughout the time course indicate points of exploration for potential intervention to mediate infection and ART-associated metabolic comorbidities.

The genus *Treponema* degrades fiber and positively correlates with high-fiber diets prevalent in rural populations (63). Tanes et al. (64) observed that a loss of *Treponema succinifaciens* significantly correlates with increased levels of circulating cytokines and MT biomarkers, including LBP and sCD14. We observed a similar trend as well, wherein lean animals exhibited a greater relative abundance of the *Treponema* genus compared to obese animals at all time points except during long-term ART at 43 weeks post-infection, which coincided with significant increases in LBP and sCD14 in lean animals only. *Prevotellaceae UCG 001* has also been shown to increase with various fiber supplementations (10). Both lean and obese animals exhibited a decrease in this genus during acute SIV infection that returned to baseline levels during ART. *Olsenella*, a genus of the Actinobacteria phylum, increased in both lean and obese animals during early ART at 25 weeks post-infection and has also been shown to increase in the gut microbiome with health-promoting diets and *Prevotella*-rich fiber-degrading microbial signatures (65, 66). The role of genera associated with high-fiber diets in microbiota-mediated crosstalk during SIV infection and viral suppression with ART should be further explored.

A longitudinal study in humans performed by Rocafort et al. (27) reported minimal changes to the gut microbiome during early HIV infection, and non-HIV-specific microbiome alterations previously associated with chronic inflammation and metabolic disorders during chronic HIV infection, including the depletion of *Akkermansia*, *Anaerovibrio*, *Bifidobacterium*, and *Clostridium*. Similar to Rocafort et al., but in contrast to previous reports (67, 68), we did not observe any significant alterations to the Firmicutes:Bacteroidetes ratio via increased Firmicutes and/or depleted Bacteroidetes at the phylum level, significant increases in the potentially harmful Proteobacteria phylum, or notable shifts from *Prevotella* to *Bacteroides* dominance. No significant differential abundances of the Firmicutes and Bacteroidetes phyla were detected in lean or obese animals throughout the time course. However, bacterial genera belonging to the Bacteroidetes phylum that significantly changed in both lean and obese animals were decreased relative to baseline. We speculate that an increase in this phylum during acute infection before beginning ART may not have occurred due to the brief time prior to ART initiation. We observed mixed trends regarding increasing and decreasing relative abundances of genera belonging to the Firmicutes phylum during acute SIV infection and throughout ART.

*Sutterella*, a genus belonging to the Proteobacteria phylum, degrades immunoglobulin A, which may contribute to the degradation of the gut barrier and translocation of microbial products, such as LPS (69). Lack of significant increases in inflammatory markers throughout infection and ART in obese animals could relate to observed decreases in *Sutterella* in obese animals. Interestingly, *Sutterella* was more abundant in lean animals during ART, which may correspond with the observed increase in serum LBP beginning at 25 weeks post-infection and eventual increases in plasma CRP and sCD14. Brown et al. (70) found that elevated CRP correlates with worsening glucose homeostasis and reduced microbial diversity. As we described previously (54), both lean and obese animals exhibited improved hemoglobin A1c levels and insulin sensitivity following SIV infection and ART, likely due to initial weight maintenance in lean animals and weight loss in obese animals. Taking these observations into account, the observed increase in CRP in lean animals was likely due to SIV infection and/or ART administration, the latter of which has previously been associated with increased levels of CRP in HIV-infected individuals on ART when compared to ART-naïve HIV-infected subjects and uninfected controls (71).

The SCFAs succinate, propionate, acetate, and butyrate can also regulate glucose homeostasis by acting as substrates for intestinal gluconeogenesis (72). Some evidence suggests that succinate produced via microbial fiber fermentation has been identified as a substrate for intestinal gluconeogenesis, and increased succinate within the gut may play a role in improving glucose tolerance (73). Other studies indicate that elevated succinate may positively correlate with impaired glucose homeostasis or play a role in the development or severity of conditions such as obesity and cardiovascular disease (74). The relative abundance of succinate-producing *Faecalibacterium* and *Bacteroides*, as well as succinate consumer *Dialister*, was higher in obese animals compared to lean animals at baseline. Succinate producers *Megasphaera* and *Ruminococcaceae* increased at 43 weeks post-infection in obese animals. In lean animals, short-term ART corresponded with an increased abundance of the succinate consumer *Dialister*. When glucose is sensed in the portal vein via the periportal nervous system, satiety signals are sent to the brain, which result in signals to decrease hepatic gluconeogenesis, therefore aiming to regulate systemic glucose levels (72, 73). By acting as an agonist for free fatty acid receptor three in the portal vein, propionate signaling affects anorectic hormones peptide YY and glucagon-like peptide 1, suggesting a link between SCFA-producing gut microbiota, satiety, energy homeostasis, and food intake (75, 76). Obese animals had a greater relative abundance of propionate-producing *Bacteroides* than lean animals at baseline and during acute SIV infection, but any significant differential abundance of *Bacteroides* between lean and obese animals was lost during ART. In obese animals, the relative abundance of Bacteroides significantly decreased from baseline during long-term ART at 43 weeks post-infection. Although these bacteria produce/consume succinate and propionate, we cannot directly infer that their relative abundances correlate with circulating SCFA levels, particularly because succinate is also a precursor to other SCFAs, butyrate, propionate, and acetate.

We attribute the observed differences between lean and obese animals mainly to established obesity and metabolic dysfunction but cannot rigorously rule out effects of the WSD independent of obesity. Overall, lean animals exhibited significant alterations in markers of inflammation and MT without evidence of significant alterations regarding microbiome alterations throughout SIV infection and ART. In contrast, obese animals exhibited insignificant longitudinal alterations in markers of inflammation and MT, which were elevated at baseline, but significant alterations in microbiome diversity and composition throughout SIV infection and ART. Unexpectedly, lean animals did not exhibit a distinct correlation between microbiome changes and global inflammation. In the same cohort of animals discussed here, we previously evaluated localized inflammation in the ascending colon via myeloperoxidase staining to assess the level of neutrophil infiltration in the intestinal barrier. Both lean and obese individuals exhibited similar levels of neutrophil infiltration at baseline that increased during acute SIV infection and subsequently decreased after treatment with ART (54). This suggests the need to look further into gut barrier integrity and localized inflammation in gut tissue to assess any direct relationships between microbiome-mediated immune activation throughout SIV infection and ART. Our data suggests that pre-existing obesity and chronic inflammation may play a factor in one’s ability to maintain microbial diversity, but that the gut microbiota may not greatly influence the development of systemic inflammation throughout SIV infection and ART.

## MATERIALS AND METHODS

### Animal diet and demographics

All data collected and analyzed were generated from a subset of SIV-infected, ART-treated lean and obese adult male rhesus macaques (*Macaca mulatta*) that have been previously described (54). Briefly, lean animals (*n* = 5) were fed a control diet providing 15% of calories from fat, 26% of calories from protein, and 59% of calories from carbohydrates (Purina Mills Monkey Diet #5000), while obese animals (*n* = 5) were fed a WSD providing 36% of calories from fat, 19% of calories from protein, and 45% of calories from carbohydrates (Purina Mills Monkey Diet #5L0P). Animal demographics are presented in Table 1 (body fat percentage determined via dual X-ray absorptiometry [DEXA] scans). The obese animals had been on the WSD for at least 5 years and exhibited a consistent obese, insulin-resistant phenotype prior to SIV infection.

**TABLE 1:** Animal demographics^*a*^

	Lean (*n* = 5)	Obese (*n* = 5)	*P* value
Age (years)	14.0 ± 1.69	15.5 ± 0.61	NS
Weight (kg)	10.23 ± 1.48	15.09 ± 1.53	<0.0001
Body fat (%)	16.96 ± 5.97	32.53 ± 6.06	<0.01

^*a*^ Body fat percentage determined using DEXA scans. Statistical significance was determined using unpaired *t*-tests.

### Viral infection, ART, and experimental timeline

Following baseline procedures performed at 4 weeks pre-infection, all animals were infected intravenously with a single high-dose, barcoded SIVmac239M strain that resulted in peak viremia at 2 weeks post-infection. A daily ART regimen comprised of tenofovir disoproxil fumarate, emtricitabine, and dolutegravir was initiated at 5 weeks post-infection and continued until necropsy at 78 weeks post-infection. Weekly blood samples were collected throughout the study to monitor viral load as previously described (54).

### Sample collection and processing

The experimental timeline is shown in Fig. 1. Serum and plasma samples were collected at the indicated time points (blue circles on timeline) and stored at −80°C. Plasma samples were analyzed for CRP and soluble CD14 (sCD14) using enzyme-linked immunosorbent assay (ELISA) kits from ALPCO (cat# 30-9710S, Salem, NH) and R&D Systems (cat# DC140, Minneapolis, MN), respectively. Serum samples were analyzed for LBP using the ELISA kit from Invitrogen (cat# EH297RBl, Waltham, MA). Feces were collected at the indicated time points (green diamond on Fig. 1 timeline) and stored at −80°C until DNA extraction. Fecal microbiome analysis was performed using 16S rRNA gene amplicon sequencing and the Qiime2 bioinformatic pipeline.

### 16S rRNA amplicon sequencing

DNA was extracted from feces using the DNEasy PowerSoil Pro Isolation Kit (Qiagen, Valencia, CA, USA Catalog #47016). The hypervariable V4–V5 region of the 16s rRNA gene was amplified using PCR primers 515F/806R, with the forward primer including a 12-base pair barcode. PCR reactions were run in duplicate, then combined, each containing 12.5 µL GoTaq master mix, 9.5 µL nuclease-free water, 1 µL template DNA, and 1 µL of 10 µM primer mixture. The thermal cycling parameters were 94°C for 3 minutes, 37 cycles of 94°C for 45 seconds, 50°C for 1 minute, 72°C for 1 minute, and a final hold at 72°C for 5 minutes. A gel of PCR product samples was run to ensure that the correct PCR fragment size was obtained. PCR products were then quantified using the Quant-IT PicoGreen dsDNA Assay Kit (ThermoFisher, catalog #P11496). Lastly, samples were multiplexed and sequenced using the Illumina MiSeq v3 kit (2 × 300 base pairs).

### 16S rRNA bioinformatic analysis

Sequence FASTQ files were processed using the Qiime2 microbiome bioinformatics platform (77). Specifically, sequences were demultiplexed and then filtered for quality using the DADA2 plugin. DADA2 filters chimeric sequences while correcting amplicon errors and generates a table of ASVs, also providing data on ASV richness as a measure of alpha diversity. Taxonomy was then assigned to the ASVs using the Qiime2 Feature Classifier compared to the Silva Database (release number 138). Sequences were aligned using MAFFT, and a phylogenetic tree was constructed using FastTree. Sequences were then rarefied to a sequencing depth of 10,000 sequences per sample prior to alpha and beta diversity analyses. Qiime2 was used to generate alpha diversity (observed ASVs) and beta diversity metrics estimated using unweighted and weighted UniFrac distances (78).

### Statistics

For CRP, LBP, sCD14, and ASV data, statistical analyses were performed using GraphPad Prism version 10.3.1 (GraphPad Software, Boston, MA). See figure legends for details regarding which statistical tests were performed. Qiime2 was used to calculate alpha diversity metrics, observed ASVs, and beta diversity measures; unweighted/weighted UniFrac distances. PERMANOVAs were performed using the Qiime diversity adonis function in Qiime2, which utilizes the Vegan R function ADONIS. The LEfSe algorithm was used to identify differentially abundant taxonomic features both between and within groups throughout the time course with a logarithmic linear discriminant analysis (LDA) score cutoff of 2 (55). ANCOM-BC was also performed using the Qiime2 composition plugin to assess differential abundance (79).

## Data Availability

16S rRNA gene sequencing data are deposited in the NCBI Sequence Read Archive (SRA) under BioProject accession number PRJNA1234568.

## SUPPLEMENTAL MATERIAL

Supplemental figures spectrum.00733-25-s0001.docx Fig. S1 to S4.

ASM does not own the copyrights to Supplemental Material that may be linked to, or accessed through, an article. The authors have granted ASM a non-exclusive, world-wide license to publish the Supplemental Material files. Please contact the corresponding author directly for reuse.
