# Supplementary material for: Impact of obesity on the gut microbiome and inflammatory markers during SIV infection and antiretroviral therapy
Source: Microbiol Spectr. 2025 Aug 27;13(10):e00733-25. doi: 10.1128/spectrum.00733-25 (PMC12502777; doi:10.1128/spectrum.00733-25)
Supplement: Supplemental figures — Fig. S1 to S4. [file spectrum.00733-25-s0001.docx]

**Impact of Obesity on the Microbiome and Inflammatory Markers during SIV Infection Treated with Antiretroviral Therapy**

**Casey M. McGuire^1^, Isaac R. Cinco^2^, Diana Takahashi^1^, Kristin A. Sauter^1^, Melissa Kirigiti^1^, Ilhem Messaoudi^2^, Jonah B. Sacha^3^, Charles T. Roberts^1,4^, and *Paul Kievit^1^**

^1^Division of Metabolic Health and Disease, Oregon National Primate Research Center (ONPRC), Beaverton, OR, USA; ^2^Department of Microbiology, Immunology, and Molecular Genetics, University of Kentucky; ^3^Division of Pathobiology and Immunology, ONPRC; ^4^Division of Reproductive and Developmental Sciences, ONPRC.

This PDF file includes:

Supplemental Figures 1-4


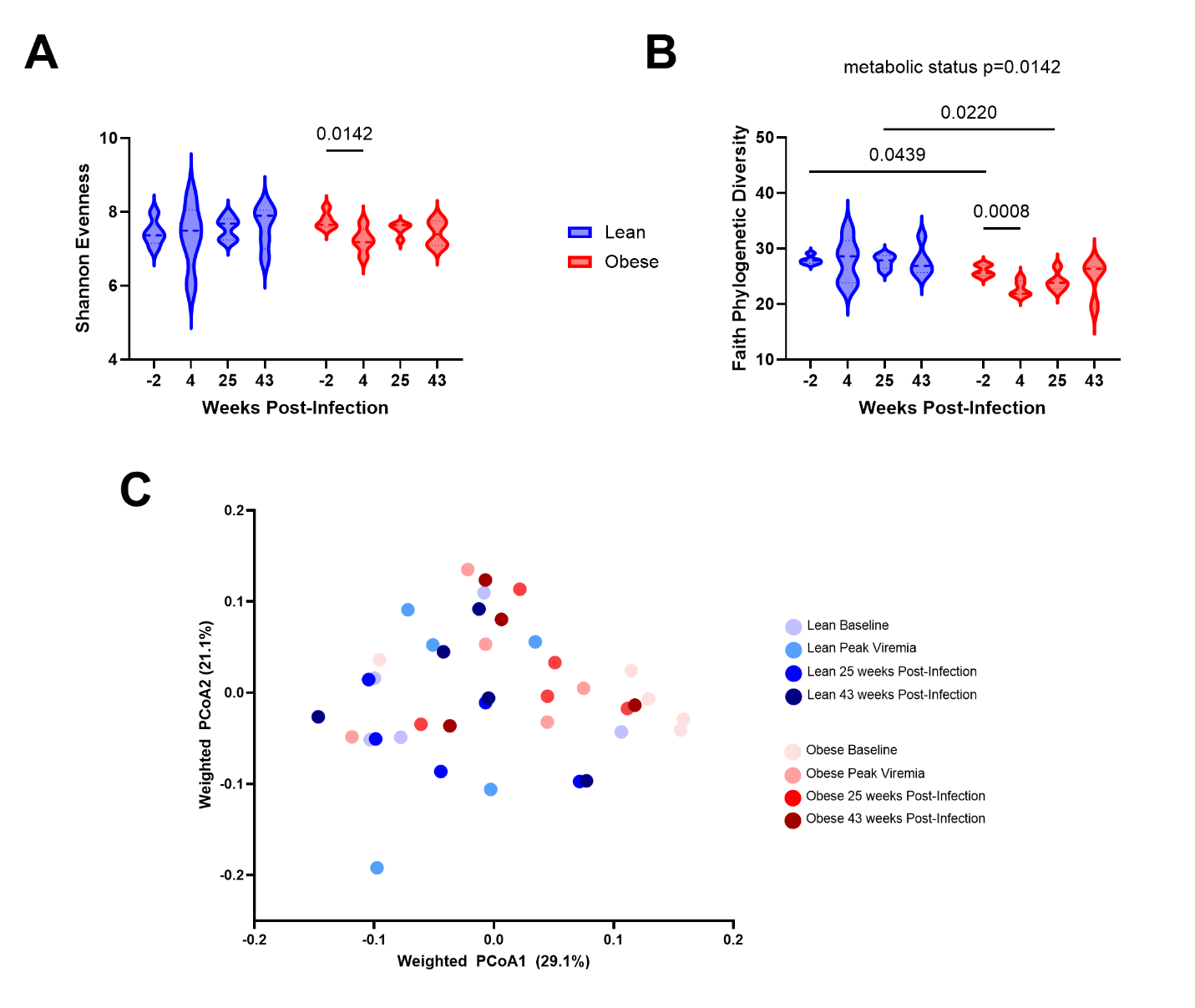


Supplemental Figure 1. **Alpha and Beta Diversity Metrics Throughout SIV Infection and ART.** Violin plots of (A) Shannon Evenness and (B) Faith Phylogenetic Diversity. After passing a normality/lognormality test, ASV significance was determined using a mixed-effects analysis with Šídák’s multiple comparisons test (between groups) and Tukey’s multiple comparisons test (within groups) (A, B). Principal coordinate analysis (PCoA) of weighted UniFrac distances between microbial communities in lean and obese animals throughout SIV infection and ART (C).


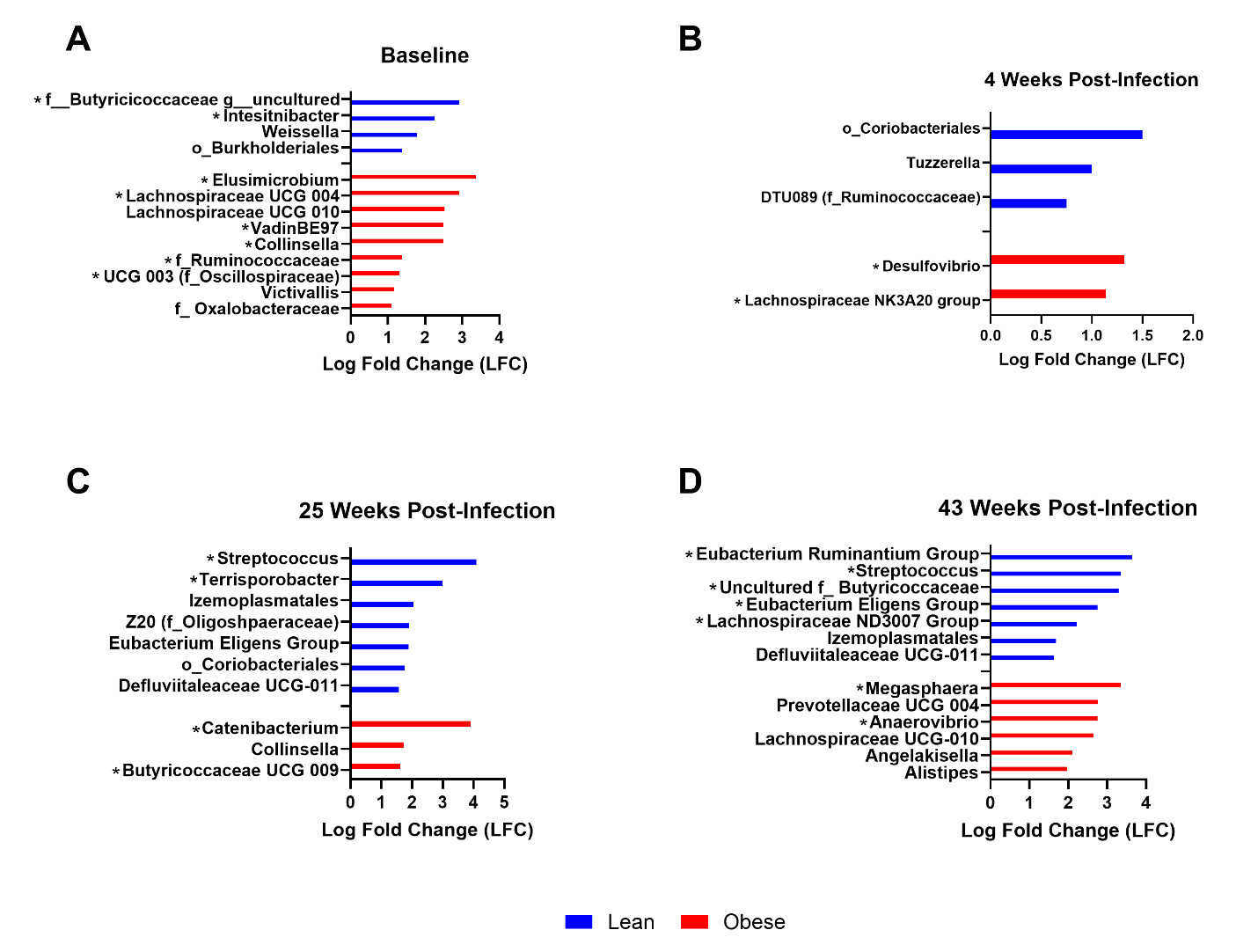


Supplemental Figure 2. **Differentially abundant genera between lean and obese animals throughout SIV infection and ART determined by ANCOM-BC analysis.** ANCOM-BC bar plots demonstrating differentially abundant genera between lean and obese animals at baseline (week -4) (A), 4 weeks post-infection (B), 25 weeks post-infection (C), and 43 weeks post-infection (D). Significant features were chosen based on a p-value less than 0.05. Taxa marked with an asterisk (*) were found to be differentially abundant in both ANCOM-BC and LEfSe analyses at one or more timepoints.


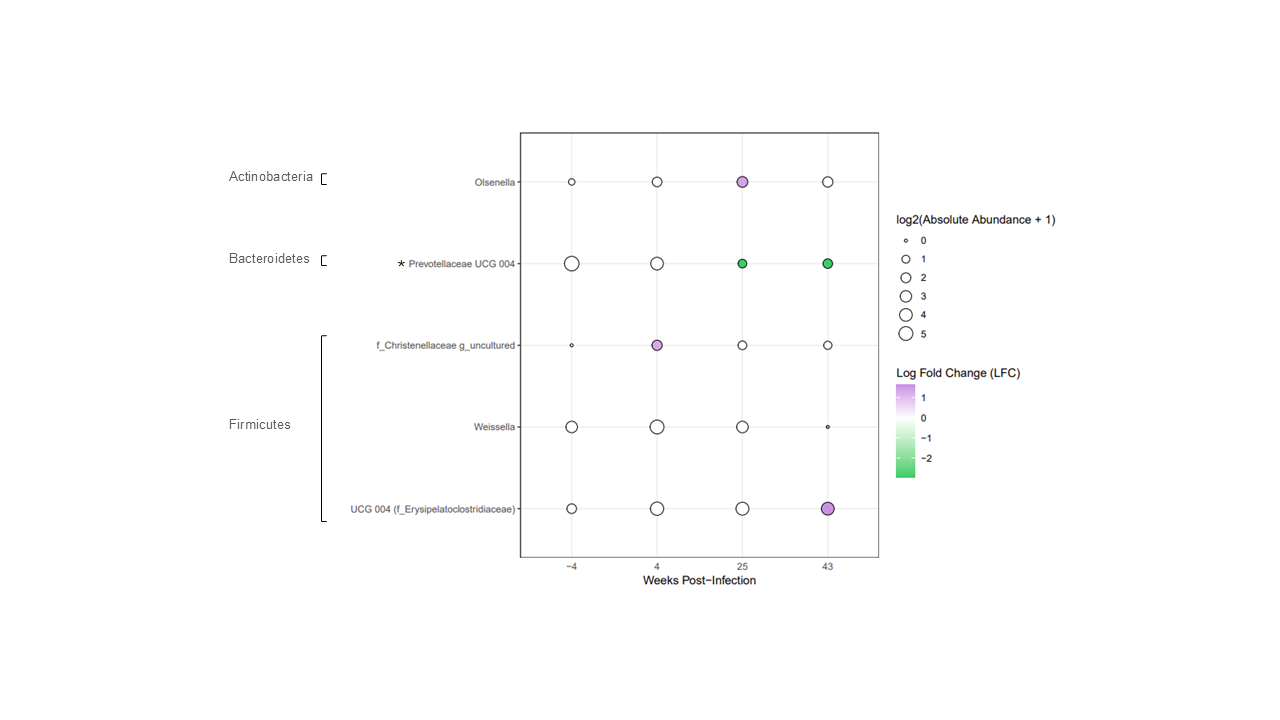


Supplemental Figure 3: **Differentially abundant genera in lean animals throughout SIV infection and ART determined by ANCOM-BC analysis**. Bubble plot of ANCOM-BC results illustrating differentially abundant genera in lean animals at 4, 25, and 43 weeks post-infection when compared to baseline (week -4). Taxa marked with an asterisk (*) were found to be differentially abundant by both ANCOM-BC and LEfSe analyses at one or more timepoints.


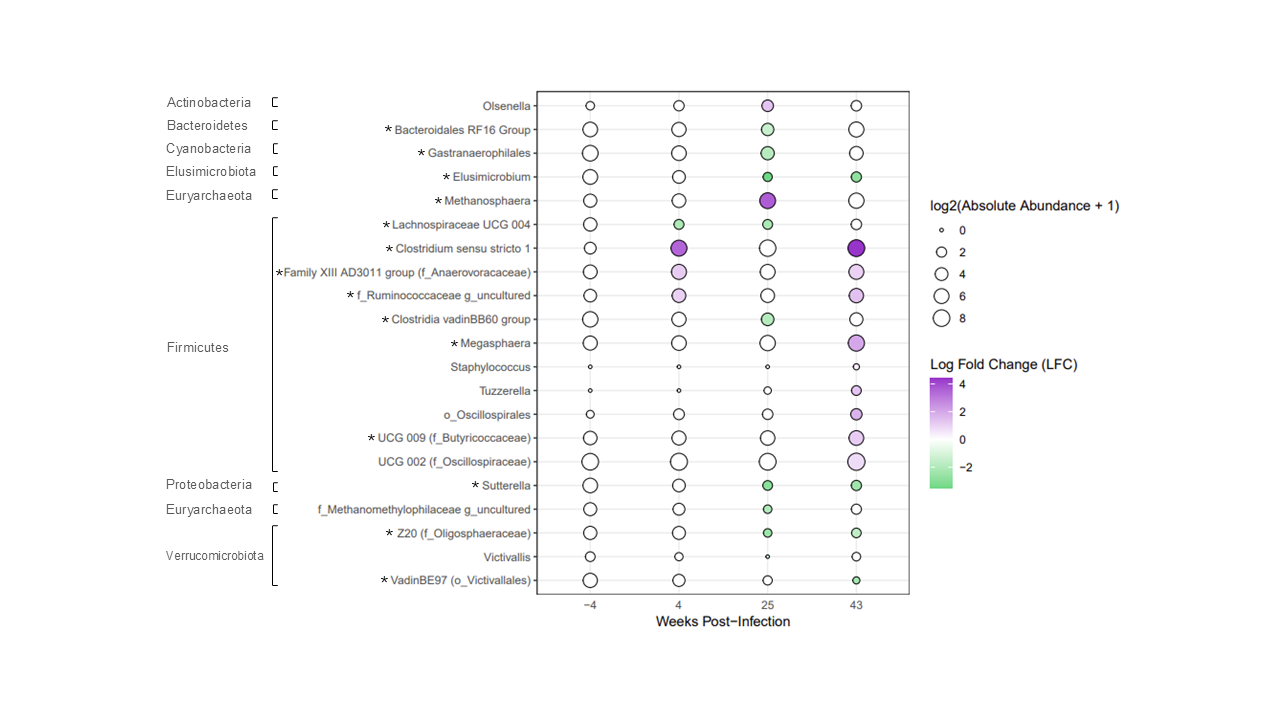


Supplemental Figure 4: **Differentially abundant genera in obese animals throughout SIV infection and ART determined by ANCOM-BC analysis**. Bubble plot of ANCOM-BC results determining differentially abundant genera in obese animals at 4, 25, and 43 weeks post-infection when compared to baseline (week -4). Bacterial phyla are indicated by the labeled brackets. Taxa marked with an asterisk (*) were found to be differentially abundant by both ANCOM-BC and LEfSe analyses at one or more timepoints.
